# Chickpea (*Cicer arietinum L*.) growth, nodulation, and yield as affected by varieties, *Mesorhizobium* strains, and NPSB fertilizer in Southern Ethiopia

**DOI:** 10.3389/fpls.2024.1372082

**Published:** 2024-04-24

**Authors:** Gashaw Nahusenay, Girma Wolde, Wondwosen Tena, Tatek Tamiru

**Affiliations:** ^1^ Department of Plant Sciences, College of Agriculture and Natural Resource, Wolkite University, Wolkite, Ethiopia; ^2^ Department of Plant Biology and Biodiversity Management, College of Natural and Computational Sciences, Addis Abeba University, Addis Abeba, Ethiopia

**Keywords:** CP-M41, CP-EAL029, CP-M20b, blended fertilizers, inoculation, cultivars

## Abstract

A significant legume crop in Ethiopia, chickpeas (*Cicer arietinum L*.) have several advantages, including high nutritional value and the capacity to improve soils deficient in nitrogen through biological nitrogen fixation using several endosymbiotic *Mesorhizobium* spp. strains. However, the host variety, the soil’s capacity to hold nutrients, and the endosymbiont’s innate physiological traits all affect how efficient the strains are. The primary obstacles to its cultivation in the research area are inadequate agronomic methods and low soil fertility [low nitrogen (N), low soil organic matter (OM), low accessible phosphorous (P), sulfur (S), and boron (B)], which results in ineffective nodulation. To evaluate the effects of NPSB fertilization and inoculation, a field experiment was carried out in Buchach Kebele’s Cheha area during the primary cropping season of 2021/22. The trial included two chickpea kinds (Local and Arerti), two NPSB levels (zero and 121 kg NPSB ha^-1^), and four levels of *Mesorhizobium* strains (CP-M41, CP-EAL 029, CP-M20b, and un-inoculated control). A randomized complete block design (RCBD) was used to organize the treatments in a factorial form with three replications. In comparison to the single application and the control, the interaction impact of strains, NPSB fertilizer, and variety greatly increased nodulation parameters, growth parameters, yield, and yield components. The Arerti variety combined with the CP-M41 *Mesorhizobium* strain and NPSB fertilizer had the maximum grain production (3177.16 kg ha^-1^). It yielded 15.96%, 24.06%, and 37.93% more than the Arerti with CP-M41 strain, Arerti with NPSB, and the control treatments, respectively. The partial budget analysis of the study treatments showed that the Arerti variety with the combined application of 121 kg NPSB ha-1 and *Mesorhizobium* strain CP-M41 inoculation produced the highest net return (102,092.6 ETB ha^-1^) with an acceptable marginal rate of return (618%). It has been found that the CP-M41 strain and the Arerti variety, when combined with 121 kg NPSB ha^-1^ application, is a suitable treatment combination to achieve increased chickpea crop yield and profit in the studied area. However, the results need further validation in the farmer’s field before recommending to farmers.

## 1 Introduction

Among grain legumes worldwide, chickpeas (*Cicer arietinum L*.) are an old crop with substantial economic value; in terms of productivity, they come in third, while in terms of harvested area, they come in second (Zehara et al., 2020). It comes in third place in Ethiopia for both production and land dedicated to legume crops. The grain is grown for food in practically every part of the nation (CSA, 2021). Chickpeas not only contribute to Ethiopia’s food security but also significantly enhance the country’s nutritional security by offering critical amino acids, fiber, protein, fat, vitamins, and carbohydrates (Gowda et al., 2016). Crop rotation with chickpea crops plays a vital part in increasing the crop’s yield and that of subsequent crops as it improves the nitrogen (N) content of soil through symbiotic nitrogen (N_2_) fixation (Zehara et al., 2020).

In Ethiopia, area coverage and total output of the Kabuli and Desi varieties of chickpeas during the cropping season of 2020–2021 were about 220, 719 ha and 4,573,193 quintals (CSA, 2021). The crop is widely cultivated and used, but its average national output under farmer’s conditions is just 2 t ha^-1^ (CSA, 2021), significantly less than its average potential yield under enhanced *management* conditions (5 t ha^-1^) (Gemechu et al., 2011). There are several reasons for the low crop productivity., including poor soil fertility (Lijalem et al., 2020), lack of nutrient supply (Endalkachew et al., 2018), lack or insufficient availability of effective indigenous native *Rhizobia* populations in soils commonly used for cereal cultivation (Endalkachew et al., 2018), which in turn led to lower biological N_2_ fixation (BNF) by the crop (Das et al., 2016), and shortage of improved varieties (Sheleme et al., 2015).

According to reports, the majority of Ethiopia’s agricultural soils are lacking in boron (B) and sulfur (S) in addition to nitrogen (N) and phosphorous (P) deficiencies (EthioSIS, 2016). Soil erosion, intensive cultivation over a long period, and minimal or unbalanced fertilizer application such as NP fertilizer (among others) are reported to be the major causes of poor soil fertility in the country (Endalkachew et al, 2018). Yield reduction in chickpeas due to deficiency of these nutrients has been reported in Ethiopia (Lijalem et al., 2020). On the other hand, several authors, such as (Kiros and Atsede, 2020), showed that applying inorganic fertilizers containing N, P, S, and B to chickpeas significantly affected their yield output. Furthermore, while it is true that chickpeas can fix 60–80% of the nitrogen they need (Giller, 2001), the true efficacy of the symbiotic relationship is probably going to vary depending on the genotypes of the *Rhizobia* strain (G_R_), the legume genotypes (G_L_), and their combination (G_L_ x G_R_) (Ashenafi et al., 2020).

One of the regions in the Gurage Zone of the Southern Nations, Nationalities, and Peoples’ Region (SNNPR) with an ideal climate for chickpea farming is the Cheha district, which is the research area. The most common type of soil in the region is vetisol. In addition to growing it for food and profit, farmers also rotate it with cereal crops. According to the soil fertility map of SNNRS developed by (ATA, 2016), the soils in the research area have low ratings for N and S and very low ratings for P and B, which goes against the crop demand. In Ethiopia generally and the research region specifically, to reduce the negative impact of poor soil fertility on crop yield, the recommended rate of combined NPSB fertilizer for food legumes is 121 kg ha^-1^ (ATA, 2016). However, because they generally believe that chickpea cultivation does not require the use of fertilizer, the majority of farmers in the study area do not use any external inputs (Cheha District Office of Agriculture, October 2021) and fertilizer is costly (Endalkachew et al., 2018). Consequently, the research area’s chickpea productivity is lower than the national average (CSA, 2021).

Hence, enhancing the N-content of soil can be accomplished more affordably by using symbiotic N_2_-fixation. To our knowledge, chickpea cultivars that yield large quantities and react well to inorganic fertilizer applications and *Mesorhizobium* inoculants were not identified for the Cheha district though different chickpea varieties are easily available in the area. In this study, it was hypothesized that the application of the recommended rate of blended NPSB fertilizer integrated with a suitable and effective *Mesorhizobium* strain would enhance the growth and yield of the crop varieties in the study area.

Thus, the goals of this study were to assess how nodulation, growth, yield, and yield components of chickpea (*Cicer arietinum* L.) were affected by *Mesorhizobium* strains, NPSB fertilizer treatment, and cultivars; to identify the suitable combination of *Mesorhizobium* inoculum, NPSB fertilizer, and variety that could improve productivity in the research area, and to assess the economic viability of using *Mesorhizobium* inoculation, NPSB fertilization and variety for chickpea production at Cheha district.

## 2 Methodology

### 2.1 Overview of the research area

**Figure 1:**
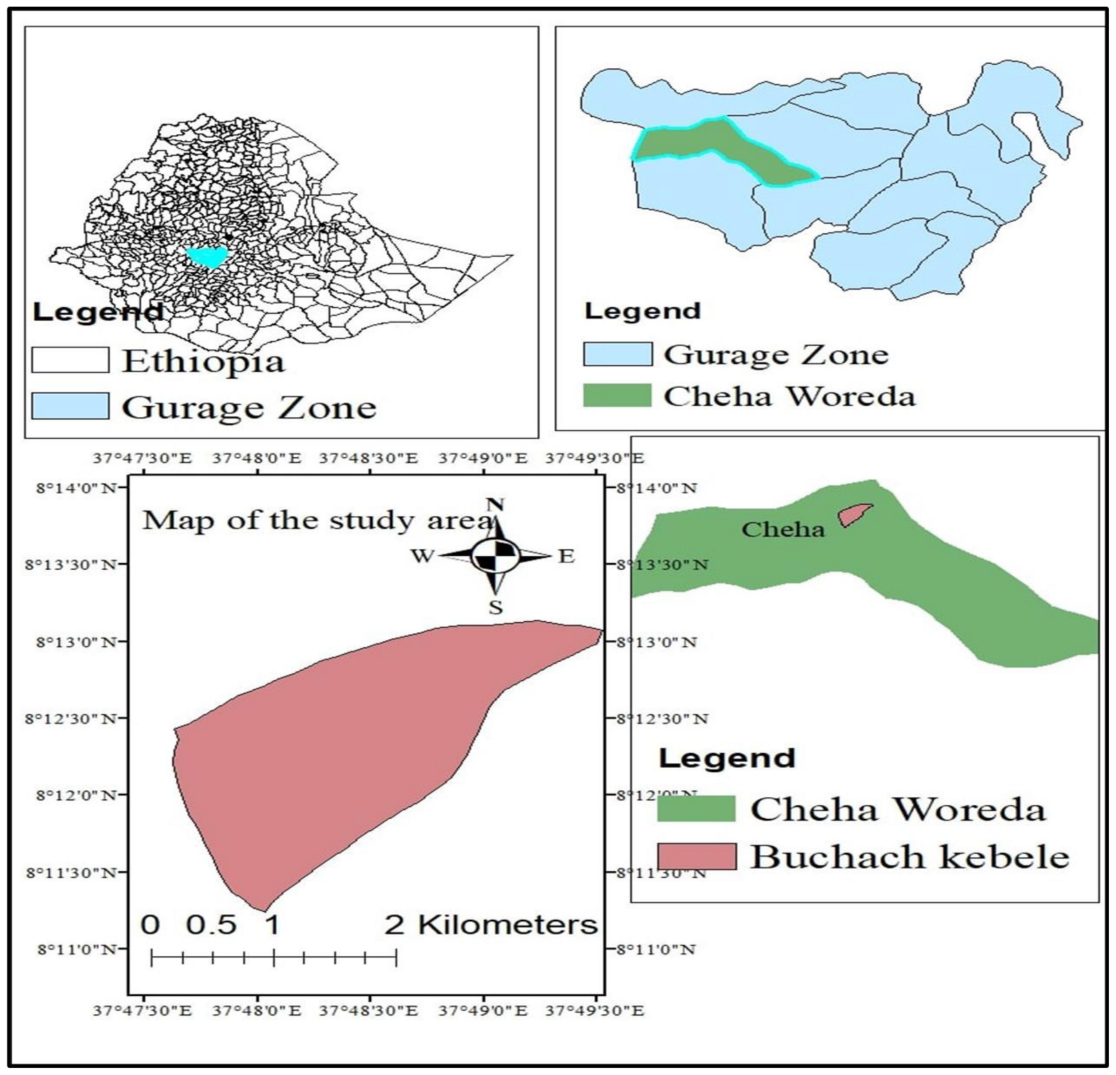
Location map of the study area.

The field study was carried out in the Cheha district’s Buchach kebele. Within the Southern Nations, Nationalities, and Peoples’ Region (SNNPR), Ethiopia, Cheha is one of the districts that make up the Gurage Zone. The site is situated 15 kilometers to the southeast of Wolkite City, the seat of the zone, and 170 km to the southwest of Addis Ababa, the capital of the country. The experimental site’s coordinates are 80 12’54’’ N longitude and 370 48’30’’ E latitude, with an elevation of 1929 meters above sea level. There are three different types of agroecology in the district: 9% is dry land (kola), 71% is mid-land (Woina dega), and 20% is high land (Dega) (**Figure 1**) (Alemayehu, 2015).

**Figure 2:**
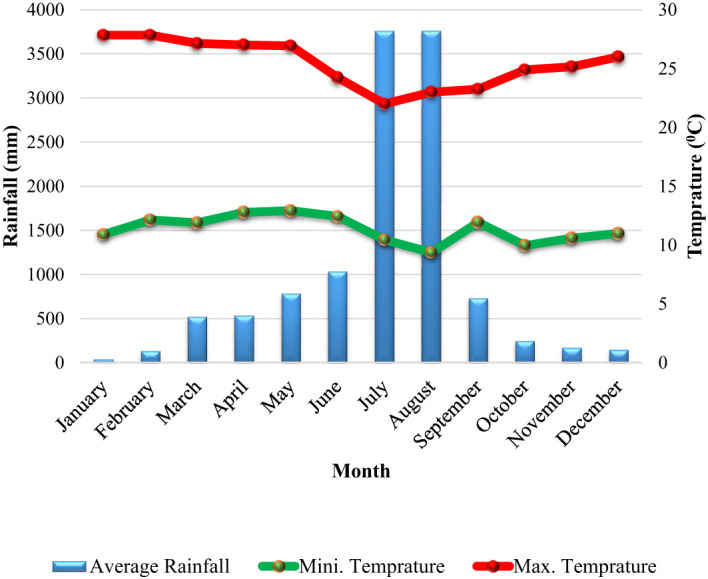
The mean monthly rainfalls (mm), maximum and minimum temperatures (^0^C) of Cheha district for ten years (2012-2021).

The main sources of income in the district are trade related to agribusiness and agriculture. The district’s predominant soil type is vetisol (Alemayehu, 2015). Enset is the main crop used in agricultural production, along with a few other crops like kale, yams, chickpeas, wheat, and barley. The principal cash crops are coffee, niger seed, teff, and khat (CWANRO (Cheha Woreda Agriculture and Natural Resource Office), 2020)^1^. Based on ten years of metrological data (2012–2022) from the Hawassa Metrological Center, the region has an unimodal rainfall pattern with an average yearly rainfall of 986.24 mm. The average monthly high and low temperatures are, respectively, 25.44 and 11.35°C (**Figure 2**).

### 2.2 Sampling, preparing, and analyzing soil

Before planting, a composite soil sample was prepared by using an auger to collect sub-samples from 20 sampling points over the entire experimental site down to a depth of 0–30 cm. This was done following the zigzag sampling approach, which allowed for the analysis of the soil’s physicochemical properties. Except for organic carbon (OC) and total nitrogen (TN), which were passed through a 0.5 mm sieve, the composite sample was air dried, ground using mortal, and thoroughly mixed before being run through a 2 mm filter for the majority of the parameters. The soil laboratory at Wolkite University and Wolkite City, Ethiopia, were used to assess the particle size distribution, pH, and accessible phosphrous (P), organic carbon (OC), and cation exchange capacity (CEC). Kjeldhal N, available Sulphur (S), and extractable boron (B) were examined in the Areka Agricultural Research Center’s soil laboratory. After drying the soil samples to consistent weights in an oven at 105°C, the bulk density of the soil was calculated using the undisturbed core sampling method. We used the hydrometer method to determine the size distribution of soil particles (Day, 1965). A digital pH meter was used to measure the pH of the soil potentiometrically in a supernatant suspension of distilled water to soil ratio of 1:2.5 (Van Reeuwijk, 1992).

The 1M ammonium acetate technique was used to calculate the cation exchange capacity (CEC) at pH 7 (Chapman, 1965). The content of organic carbon (OC) was ascertained using the methodology outlined by Walkley and Black (Walkley and Black, 1934).

The micro kjeldhal technique was used to assess the kjeldhal nitrogen in the soil (Jackson, 1958). Available P was examined using the Olsen technique (Olsen et al., 1954).

Using the extraction method of monocalcium phosphate, available S in the soil was determined (Hariram and Dwivedi, 1994), and the hot water method was used to determine extractable B (Berger and Truog, 1939).

### 2.3 Explanation of the test materials

*Mesorhizobium* strains CP-M41, CP-EAL 029, and CP M20b (on lignite-based carrier) used as inoculant bio-fertilizer were collected from Menagesha Biotechnology Industry Private Limited Company (MBI PLC), Addis Ababa. These *Mesorhizobium* strains were selected based on their symbiotic effectiveness in chickpeas and their ability to enhance chickpea yield under wide ecological conditions in Ethiopia (Wondwosen et al., 2016; Zehara et al., 2020). The improved chickpea variety Arerti (Kabuli type), and the local variety (Desi type) were used in this experiment. The seeds of the improved variety were received from the Debre Zeit Agricultural Research Center (DZARC), Ethiopia, and we got the farmers’ seeds of the local type. The improved Arerti variety, representing the Kabuli type, was selected because of its better yield, market preference, adaptability, and widespread cultivation by smallholder farmers. The blended fertilizer NPSB (18.9 N – 37.7 P_2_O_5_ – 0 K_2_O - 6.95 S - 0.1 B) was used (ATA, 2016), and obtained from the Plant Science Department, Wolkite University, Wolkite, Ethiopia.

### 2.4 Experimental treatments, design, and layout

A three-factor experiment was conducted in this investigation. Two chickpea types (local and Arerti), four inoculation levels (un-inoculated, CP-M41, CP EAL 029, and CP M20b), and two NPSB fertilizer kinds (without, 0 kg ha^-1^, and with 121 kg ha^-1^) were arranged in a factorial combination using a factorial randomized complete block design (RCBD) with three replications. Sixteen treatment combinations were used in the investigation. The sixteen treatment combinations were represented by the sixteen plots in each replication. A total of 48 plots having a dimension of 2 m × 2.4 m (4.8 m^2^) were prepared. The plots were kept 1 m apart and the spacing between blocks was 1.5 m to minimize contamination (Abere et al., 2019). Following the specification of the design, treatments within the block were assigned randomly to experimental units using the lottery method. 30 cm between each seed and 10 cm between each row were used for seeding (ATA, 2007), having 8 rows per plot. In every plot, there were 160 plants in total.

### 2.5 Experimental protocols and field operations

The trial location was chosen and prepared with the following criteria: no history of inoculation, cereal crops the season before, a level slope (4%), and no standing water. The experimental field had been well-plowed and leveled before planting. To prepare the field for planting, plowing was carried out following the crop’s suggestion and according to standard procedure. According to the experimental plan, the land was leveled and split into small plots and blocks. A field layout was created based on the design.

According to Abere et al. (2019), seed inoculation was performed using the procedure developed by Fatima et al. (2007). The corresponding rhizobial strains were injected into the seeds right before planting. Chickpea inoculation was carried out using the suggested lignite-based carrier-rhizobial biofertilizers at 500 g ha^-1^ as advised by Abere et al. (2019). To guarantee that the applied inoculant adhered to the seeds, the necessary amount of seed was suspended in a 10% sugar solution at a 1:1 ratio. At the rate of 10 g kg^-1^ of seed, the inoculant was carefully combined with dry seeds.

To preserve the viability of the cells, the inoculation was done right before seeding under shade. The seeds were then sowed into the corresponding plots at the recommended rate and spacing after being allowed to air dry for a short while.

Sowing was done by hand at about 5 cm depth on randomly allocated plots within each replication at the recommended row spacing (ATA, 2007). To avoid contamination, un-inoculated plots were sown first. To lessen fertilizer and bacteria from one plot to the next due to rain, ridges were created between each block and plot. All other agronomic techniques were implemented consistently across all plots following the crop’s recommendations (ATA, 2007).

### 2.6 Data collection and measurements

Ten randomly chosen plants from a row adjacent to the border row of each plot were used to record nodulation parameters (such as the total number, volume, and dry weight of nodules per plant) and growth characteristics (such as shoot and root dry weights) throughout the mid-flowering stage of the crop. Ten representative nodules from each of the ten uprooted plants were used to calculate the number of effective nodules per plant. The nodules were then cut open with a knife to reveal the color of the center. Phenological parameters were also recorded with corresponding times, such as days to 50% flowering and 90% physiological maturity. At maturity, growth metrics were measured from the center rows of each plot, including plant height and the number of primary and secondary branches per plant. Data on yield components, such as the number of pods per plant, the number of seeds per pod, and the weight of one hundred seeds for each plot, were recorded during the harvesting period. The net plot area (1.92 m^2^) was used to record the yield metrics, which included aboveground dry biomass yield, grain yield, straw yield, and harvest index.

### 2.7 Analysis of statistical data

Using a general linear model (GLM technique) in statistical analysis software (SAS) version 9.3, the acquired data were examined using a three-way analysis of variance (ANOVA) (SAS, 2012). The least significance difference (LSD) test was used to compare means at a 5% probability level if significant differences in the F-test were found. A simple analysis of correlation was carried out between yield, yield components, and other pertinent variables.

### 2.8 A partial analysis of the budget

The economic viability of the treatments was examined using economic analysis. The average yield from the experimental plots was deducted by 15% to account for the variation between the experimental yield and the yield that farmers could anticipate from the same treatment, or 10% for differences in management and 5% for differences in plot size (CIMMYT, 1988). Accordingly, a discrete partial budget analysis was performed using the steps described by (CIMMYT, 1988) on the mean grain yields for *Mesorhizobium* strains, NPSB fertilizer, and various treatment combinations. To calculate economic factors, the fluctuating cost of *Mesorhizobium* strains (45 Ethiopian birrs (ETB) bag^-1^ (125g)), NPSB fertilizer (18.60 ETB kg^-1^), and chickpea seeds (25 and 32 ETB ka^-1^ for local and Arerti, respectively) were made by taking into account the cost at planting (September 2021). The field price of current chickpea grain at the time of harvesting (30 and 35 ETB kg^-1^ for local and Arerti varieties, respectively) was extracted from the Cheha district’s Office of Trade and Transportation marketing team (January to February 2022). The cost of *Mesorhizobium* strains and NPSB fertilizer was obtained from Menagesha Biotechnology Industry PLC and Agricultural Inputs Supply Enterprise, respectively. Ethiopian Birr (ETB ha^-1^) was used to calculate all costs and benefits on a per-hectare basis. A therapy must have a minimal acceptable rate of return (MRR) of at least 50% to 100% for farmers to view it as a viable choice (CIMMYT, 1988).

Therefore, the study’s marginal analysis led to the farmers’ suggestions, and a 100% return on investment was deemed to be the minimum acceptable rate of return.

## 3 Results and discussion

### 3.1 Soil’s physicochemical characteristics before planting

The study area’s composite soil sample analysis revealed that the experimental site’s soil had a particle size distribution of 68% clay, 21% silt, and 11% sand. Thus, the soil texture of the experimental site was clay according to the soil study that was done. According to (Hunt and Gilkes, 1992). the soil’s bulk density fell into the moderate category at 1.323 gm/cm^3^. The soil’s pH was 5.7, indicating a moderately acidic reaction (Tekalign et al., 1991). For the majority of legume crops, including chickpeas, this pH level is ideal as it promotes biological nitrogen fixation (Jordan, 1984; Horneck et al., 2011). The experimental soil had values of 2.1%, 44.8 mol (+) ka^-1^, 0.16%, 2.69 mg kg^-1^, 6.34 mg kg^-1^, and 0.443 mg kg^-1^ (ppm) for organic carbon (OC) content, cation exchange capacity (CEC), kjeldhal nitrogen, available phosphorus, available sulfur, and extractable boron, according to the analysis of other soil chemical properties performed before sowing (**Table 1**).

**Table 1:** Physico-chemical characteristics of surface soil before planting.

Soil characters	Unit	Value	Rating	References
Bulk density	gm/cm^3^	1.323	Moderate	Hunt and Gilkes, 1992
Sand	%	11	_	_
Silt	%	21	_	_
Clay	%	68	_	_
Textural class	_	Clay	_	_
PH (H_2_0)	_	5.7	Moderately acid	Tekalign et al., 1991
Organic carbon	%	2.1	Low	Landon, 1991
CEC	cmol (+) kg^-1^	44.8	Very high	Hazelton and Murphy, 2007
Kjeldhal nitrogen	%	0.16	Low	Landon, 1991
Available P	mg kg^-1^	2.69	Very low	Olsen et al., 1954
Available S	mg kg^-1^	6.34	Low	Lewis, 1999
Extractable B	mg kg^-1^	0.443	Low	Reisenauer et al., 1973

Thus, the experimental soil’s ratings for its organic carbon (OC) content, cation exchange capacity (CEC), total nitrogen, available phosphorous, accessible sulfur, and extractable boron were low, very high, low, very low, low and low, following Olsen et al., 1954; Landon, 1991, 1991; Lewis, 1999; Hazelton and Murphy, 2007, and Reisenauer et al., 1973), respectively. The low levels of organic carbon and nitrogen in the studied area suggest that the soil is not very fertile. The findings suggest that nitrogen was a growth-limiting factor for crops in the study area, potentially as a result of ongoing cultivation and the absence of organic material incorporation. Consequently, it is imperative to reduce this growth-limiting factor by applying fertilizer containing nitrogen and/or inoculating with effective strains. One feature that most Ethiopian soils have in common is low N and P levels (Tekalign et al., 1991). The low S was predicted since the experimental soil’s low organic matter content—a source of around 95% of S—indicates a poor potential for mineralization-induced S supply to plant growth. Low micronutrients similar to this result (low B content) were reported (EthioSIS, 2016). Hence phosphorous, sulfur, and boron-containing fertilizers such as NPSB should be added to meet the crop’s needs.

### 3.2 Effects of *Mesorhizobium* strains, varieties, and NPSB fertilizer on chickpea phenological parameters

#### 3.2.1 Days to 50% flowering and 90% physiological maturity

*Mesorhizobium* strains, varieties, and NPSB fertilizer application significantly affected both days to 50% flowering and 90% physiological maturity. However, interaction effects were not significant for both parameters (**Table 2**). Mean values across the two chickpea cultivars revealed that the flowering and physiological maturity of the variety Arerti were delayed by 2.13 and 5.34 days, respectively compared to the local cultivar (**Table 3**). This may be because of the differences in their genes in response to flowering as chickpeas have very high diversity in such phenological characters (Gemechu et al., 2012). Similarly (Tamiru and Girma, 2019a), reported that the Arerti type of chickpeas required the most days to reach bloom and maturity. In line with this result (Molla, 2016), also showed a highly significant effect of chickpea cultivars on the duration of days required to attain 90% physiological maturity and 50% flowering in southern Ethiopia.Inoculation with *Mesorhizobium* strains significantly (P ≤ 0.001) delayed both days to 50% flowering and 90% physiological maturity of chickpea plants (**Table 3**). The CP EAL 029 strain and the CP M41 strain had the longest days of blooming and maturity, respectively. These results were statistically comparable to those obtained from the CP M20b strain. The plants that had not received an injection had the lowest levels for both metrics. *Rhizobial* inoculation may have delayed blooming and maturity because it promoted N fixation and thus increased plant absorption, which improved chickpea vegetative growth and postponed flowering and maturity (Admasu, 2019). This outcome concurs with the (Assefa, 2016) discovery that rhizobia-inoculated seeds lengthened the time it took for chickpeas to mature and blossom.

**Table 2:** Mean squares ANOVA for phenological and growth parameters of chickpea to seed inoculated by Mesorhizobium strains, Varieties, and NPSB fertilization.

SV	DF	DTF	DTM	PHPL	NPBPL	NSBPL	SHDWPL (g)	RDWPL (g)
**Rep**	2	0.39	24.93	63.39	0.28	3.88	2.81	3.04
**V**	1	54.18***	346.68***	93.94***	0.05***	9.99***	138.07***	1.29**
**RI**	3	8.96***	30.68**	62.58***	1.42***	27.60***	120.17***	1.28***
**NPSB**	1	9.18**	123.52***	184.27***	1.54***	58.30***	318.42***	3.47***
V*RI	3	1.63^NS^	13.68^NS^	38.61***	0.26***	4.90***	48.87***	0.2^NS^
V*NPSB	1	0.02^NS^	6.02^NS^	6.27^NS^	0.03*	0.22^NS^	1.42^NS^	0.009^NS^
RI*NPSB	3	0.85^NS^	10.41^NS^	7.59*	0.02*	1.84***	3.08*	0.21^NS^
V*RI*NPSB	3	0.24^NS^	8.35^NS^	12.96**	0.04***	1.05**	14.73***	0.07^NS^
**Error**	30	1.17	7.89	1.96	0.006	0.21	0.89	0.105
**Total**	47							

SV, Sources of variation; *Significant at p ≤ 0.05; **significant at p ≤ 0.01; ***significant at p ≤ 0.001 by LSD; NS, non-significant at p > 0.05. DF, Degree of freedom; N, nitrogen; P, phosphorus; S, Sulfur; B, boron; V, Variety; RI, *Mesorhizobium* inoculants; DTF, Days to 50% flowering; DTM, Days to 90% Physiological maturity; PHPL, Plant height per plant; NPBPL, number of primary branches per plant; NSBPL, number of secondary branches per plant; SHDWPL, Shoot dry weight per plant; RDWPL, Root dry weight per plant.

**Table 3:** Mean effects of *Mesorhizobium* inoculants, NPSB fertilizer, and varieties on days to 50% flowering (DF) and 90% physiological maturity (DM) of chickpea at Cheha district.

Treatments	DF	DM
Variety		
Local	56.70^b^	98.00^b^
Arerti	58.83^a^	103.37^a^
P value	***	***
LSD (5%)	0.63	1.65
Mesorhizobium Strains		
Un-inoculated	56.50^b^	98.41^b^
CP M41	58.17^a^	102.16^a^
CP EAL029	58.41^a^	101.16^a^
CP M20b	58.00^a^	101.00^a^
P value	***	**
LSD (5%)	0.90	2.34
NPSB Fertilization		
Without NPSB (0 kg ha^-1^)	58.20^a^	102.29^a^
With NPSB (121 kg ha^-1^)	57.33^b^	99.08^b^
P value	**	***
LSD (5%)	0.63	1.65
CV (%)	1.87	2.79

Main effect means within a column followed by the same letter(s) are not significantly different from each other at P < 0.05 according to Fisheries LSD test; NS, non-significant; ** = significant at P ≤ 0.01; *** = significant at P ≤ 0.001; LSD, Least Significance Difference; CV, Coefficient of variation.

When compared to plots treated with NPSB, chickpea plants cultivated without NPSB fertilization took longer to reach 90% physiological maturity and 50% blooming (**Table 3**). The phenomena wherein increased availability of the nutrients in fertilizers increases their uptake by the plants and fosters more rapid development and beginning of flowering may be responsible for the fastening duration of flowering and maturity in response to fertilizer treatment. Additionally, the P given by the mixed NPSB fertilizer—which has a high P and a low N rate—could be one factor in the shortening of flowering and maturity days as a result of its application. According to reports, the P-rich NPSB blended fertilizer type was crucial for crop maturity acceleration, blooming, and seed output (Admasu, 2019). Furthermore, days to flowering and maturity may be accelerated by the inclusion of micronutrient B in blended fertilizer. This suggests that B’s metabolic impact in the blended NPSB fertilizer accelerates the time to flowering and maturity. The observation that a lack of boron delays the germination of pollen and the growth of pollen tubes, ultimately stopping blooming and fruit set, was validated by this. Additionally, B stimulates P absorption, directly promoting flowering. In accordance with this outcome (Wazir et al., 2018; Admasu, 2019), revealed that the days to flowering and maturity dropped when the P rate increased. Similarly ((China Gebru, 2018)), noticed that chickpea genotypes treated with NPSZnB had shorter days for blooming and physiological maturity than the control treatment.

### 3.3 Effects of *Mesorhizobium* strains and NPSB fertilizer on nodulation parameters of chickpea varieties

#### 3.3.1 Number of nodules and nodule volume per plant

**Figure 3:**
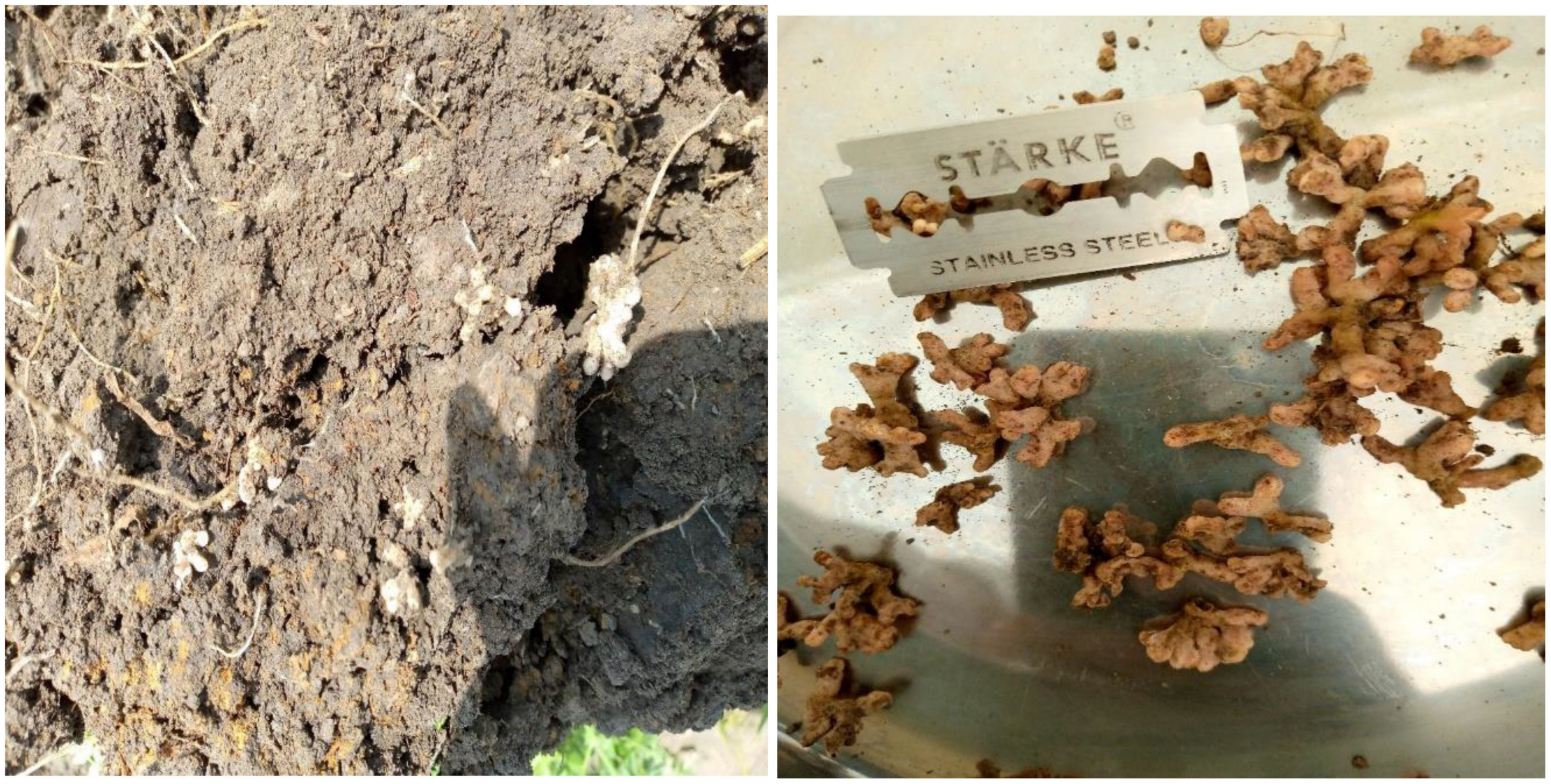
Showing the nodulation performance of inoculated chickpea plants in a plot.

The three-way interaction effects of varieties, strains, and NPSB fertilizer had a significant effect on these parameters (**Table 4**). The maximum number of nodules (19.2) and nodule volume (9.9 ml plant^-1^) per plant of chickpea was recorded in the Arerti variety with CP-M41 strain and NPSB fertilizer application followed by the use of Local variety with CP-EAL 029 strain and NPSB fertilizer application. In contrast, the lowest nodule number (10.79) and volume (4.13 ml plant^-1^) were recorded when the Local variety was grown without inoculation and fertilizer application (control treatment) (**Table 5**). Compared to the control treatment (un-inoculated and unfertilized), nodule number and volume per plant of Arerti variety were increased by 36.61 and 51.21%, respectively due to the interactive use of CP-M41 strain and NPSB fertilizer. **Figure 3** shows the nodulation performance of inoculated chickpea plants in the experimental plot.Notable numbers of nodules were also seen in the control treatments in this investigation, indicating the presence of native Rhizobia species that may produce small-sized nodules on lateral roots, the majority of which were ineffective (white) (**Table 5**). Nonetheless, the increased volume and quantity of nodules brought about by the strain inoculation indicated that there was improved synergy between the chickpea plant and the introduced Rhizobia. Conversely, strain specificity between tested and applied strains of Cultivar-Rhizobia may account for the two chickpea varieties’ differing nodulation responses to the combined application of strains and NPSB fertilizer (Ashenafi et al., 2020).

**Table 4:** Mean squares ANOVA for nodulation parameters of chickpea to seed inoculated by *Mesorhizobium* strains, Varieties, and NPSB fertilization.

Sources of variation	DF	TNNPL	ENNPL	NVPL (ml)	NDWPL (g)
**Rep**	2	2.00	0.11	0.31	0.11
**V**	1	22.68***	0.3***	5.005***	0.82***
**RI**	3	25.72***	1.02***	14.85***	1.21***
**NPSB**	1	55.47***	2.43***	29.29***	3.83***
V*RI	3	9.34***	0.59***	5.17***	0.64***
V*NPSB	1	0.27^NS^	0.003^NS^	0.13^NS^	0.037*
RI*NPSB	3	0.99*	0.026*	0.24**	0.043**
V*RI*NPSB	3	2.09***	0.14***	1.22***	0.14***
**Error**	30	0.29	0.0077	0.06	0.006
**Total**	47				

* Significant at p < 0.05; ** significant at p < 0.01; *** significant at p < 0.001 based on LSD at p ≤ 0.05; NS, non- significant at p > 0.05. DF, Degree of freedom, N, nitrogen, P, phosphorus, S, Sulfur, B, boron; V, Variety; RI, *Mesorhizobium* inoculants; TNNPL, Total number of nodules per plant; ENNPL, Effective nodule number per plant; NVPL, Nodule volume per plant; NDWP, Nodule dry weight per plant.

**Table 5:** Three-Way Interaction Effects of Variety, *Mesorhizobium* Strains, and NPSB Fertilizer on Nodulation and Growth Parameters of Chickpea in Cheha District.

Treatments			TNN	NV (ml)	NEN	NDW (g)	PH (cm)	NPB	NSB	SHDW (g)
Variety	NPSB	Strains								
Arerti	Control	Control	12.2^g^	4.8^j^	1.9^g^	2.8^i^	42.7^e^	2.4^h^	7.9^f^	34.9^hi^
		CP M41	16.4^c^	7.1^ef^	2.6^cd^	3.4^ef^	46.7^bcd^	3.0^cde^	10.4^d^	39.9^de^
		CP EAL-029	14.9^de^	6.5^g^	2.3^e^	3.1^gh^	44.9^cde^	2.8^ef^	9.8^de^	38.9^ef^
		CP M20b	15.6^cd^	7.0^ef^	2.5^de^	3.3^fg^	45.1^cde^	2.9^def^	9.9^de^	39.7^de^
	121 kg ha^-1^	Control	15.7^cd^	6.5^g^	1.9^g^	3.3^fg^	44.4^cde^	2.7^fg^	8.9^ef^	39.6^de^
		CP M41	19.2^a^	9.9^a^	3.4^a^	4.3^a^	56.4^a^	3.7^a^	14.2^a^	48.3^a^
		CP EAL-029	15.9^cd^	7.3^de^	2.5^cd^	3.6^de^	47.6^bc^	3.1^cd^	11.7^bc^	41.0^d^
		CP M20b	17.5^b^	8.4^c^	2.9^b^	3.9^c^	49.7^b^	3.3^bc^	12.6^b^	43.7^c^
Local	Control	Control	10.9^h^	4.1^k^	1.8^gh^	2.5^j^	38.1^f^	2.1^i^	6.6^g^	29.9^k^
		CP M41	14.0^ef^	5.8^h^	2.1^f^	2.9^hi^	42.7^ef^	2.8^ef^	8.8^ef^	34.6^ij^
		CP EAL-029	14.9^de^	6.8^fg^	2.5^de^	3.2^fg^	44.9^cde^	3.1^cd^	9.9^de^	37.6^fg^
		CP M20b	14.4^ef^	6.5^g^	2.3^e^	3.2^g^	43.5^def^	2.9^de^	9.3^e^	36.4^gh^
	121 kg ha^-1^	Control	13.4^f^	5.3^i^	1.9^g^	2.8^i^	42.9^def^	2.6^gh^	8.1^f^	32.9^j^
		CP M41	15.0^de^	6.9^efg^	2.5^de^	3.2^fg^	45.3^cde^	3.1^cd^	10.7^cd^	38.9^ef^
		CP EAL-029	17.9^b^	9.1^b^	3.2^a^	4.1^b^	49.7^b^	3.5^ab^	12.7^b^	45.6^b^
		CP M20b	15.9^cd^	7.7^d^	2.7^bc^	3.7^cd^	46.1^bcde^	3.1^cd^	11.8^bc^	43.1^c^
P value			***	***	***	***	**	***	**	***
CV (%)			4.14	4.01	4.8	3.47	5.25	5.19	6.53	2.58

Interaction means within a column followed by the same letter (s) are not significantly different from each other at a 5% level of significance based on the Fishers LSD test; ** = significant at P ≤ 0.01; *** = significant at P ≤ 0.001; CV, Coefficient of variation; TNN, Total Number of Nodules; NEN, Number of Effective Nodules; NV, Nodule Volume; NDW, Nodule Dry Weight; PH, Plant height; NPB, Number of the primary branch; NSB, Number of secondary branch; SHDW, Shoot dry weight.

The highest value of nodule number and volume due to the interaction of CP-M41 strain and NPSB fertilizer application may be because P, S, and B combined with inoculant play such an important role in improving nodule formation and also for the roots to become infected by the rhizobia bacteria and develop nodules (Bolanos et al., 1996; Singh et al., 2018). The legume’s native *Rhizobium* population density may not be sufficient, as seen by the increase in nodule number and volume when the seeds were treated with a *Rhizobium* strain and NPSB nutrients. This, in turn, caused the plant to respond to the *Rhizobium* strain inoculation. The highest value may be attributed, in particular, to P and B working in tandem with the CP-M41 strain to play a critical role in the consumption of S, increasing the number of nodules and facilitating the process of N fixation. Therefore, the combination of the high nitrogen fixation process carried out by P and B activity and the creation of ferredoxin by S and CP-M41 may have led to the greatest value from NPSB*CP-M41, increasing the size of nodules. This noteworthy outcome may potentially be attributable to the fact that the CP-M41 strain had better nodulation-inducing capacity than CP-EAL 029 and CP-M20b strains, and naturally pre-existing soil *Rhizobium* species. But in CP-M20b * NPSB and CP-EAL 029 * NPSB interactions, the lower performance of both CP-M20b and CP-EAL 029 strains than CP-M41 strain might result in significantly lower values of nodule number and volume than CP-M41 * NPSB strain interaction. The current outcome concurred with (Singh et al., 2018; Tamiru and Girma, 2019b; Mulugeta et al., 2018), who reported that the number of nodules in chickpea increased when inorganic fertilizers and rhizobia inoculant were applied together. In addition (Merkebu, 2019), reported that when NPSZn mixed fertilizer and the MB003 *Rhizobium* strain were applied simultaneously to the mungbean crop, the diameter of the nodules rose.

#### 3.3.2 Effective nodule counts per plant

The three-way interaction effects of cultivars, *Mesorhizobium* strains, and NPSB fertilizer application significantly (P<0.001) altered the number of effective nodules per chickpea plant (**Table 4**). When the Arerti variety was infected with the CP M41 strain and NPSB fertilizer, the maximum number of effective nodules (3.37) was recorded; however, this number was statistically equivalent to that obtained from the local variety inoculated with CP EAL-029 under NPSB application. The parameter’s lowest value was registered when the local variety was planted without inoculation and fertilizer application (**Table 5**). The nodule color due to the combined application of *Mesorhizobium* strains with NPSB fertilizer on both varieties ranged between pink and deep dark red, while white and/or green nodule color was observed on the varieties planted without inoculation and fertilizer application, which demonstrated the inefficiency of the natural *Rhizobia* in the soil. The effective nodules of chickpeas at the interactive use of *Mesorhizobium* strains with NPSB fertilizer may be a result of the beneficial effects of the ideal dosage of mineral NPSB fertilizer combined with inoculation on the development of nodules and the synthesis of leghemoglobin (Nuru, 2020). The CP-M41 *Rhizobium* strain’s aptitude for successful nodulation and the increased availability of nutrients like P, S, and B after NPSB application—all of which are essential for effective nodulation—could be contributing factors to the rise in the number of effective nodules. This could be explained by sulfur’s beneficial effects on nodulation because it’s essential for the manufacture of nitrogenase, an enzyme used in nitrogen fixation. The nodulation performance of the CP-EAL 029 and CP-M20b strains may be inferior to that of CP-M41 in NPSB*CP-EAL 029 and NPSB*CP-M20b interactions, leading to a lower value than CP-M41. This also indicated that the added fertilizer is not fulfilling all the requirements of the crops N demand. This result is in line with the findings of (Beza, 2017), who found that applying inorganic fertilizers along with *Rhizobium* inoculation enhanced the effectiveness of nodules on chickpeas.

#### 3.3.3 Nodule dry weight per plant

This metric was significantly (P<0.001) impacted by the three-way interaction effect of *Mesorhizobium* strains, cultivars, and NPSB fertilizer (**Table 4**). Among the three-way interactions, variety Arerti recorded the maximum nodular dry weight of 4.35 g plant^-1^ when inoculated with CP M41 strain and applied with 121 kg NPSB ha^-1^ followed by the local variety (4.09 g plant^-1^) inoculated with CP EAL 029 strain and applied with 121 kg NPSB ha^-1^. On the other hand, the minimum nodule dry weight was obtained from local varieties planted without inoculation and fertilizer application (**Table 5**). The reason behind the higher nodular dry weight per plant on Arerti for the interaction effects of NPSB * CP-M41 strain may be that the combination of P and S with rhizobia bacteria plays a role in improving the leghemoglobin contents of nodular tissue and increasing nitrogenase activity. This, in turn, may increase the size of nodules and ultimately their dry weight (Singh et al., 2018). This research demonstrated the significance of the nutrients (P, S, and B) that are supplied in encouraging the growth of large root systems, which raises the possibility of nodule development. A comparable finding was also noticed by (Mulugeta et al., 2018; Admasu, 2019).

### 3.4 Effects of *Mesorhizobium* strains, varieties, and NPSB fertilizer on growth parameters of chickpea

#### 3.4.1 Plant height

This parameter showed a highly significant (P<0.01) effect from the three-way interaction effects of *Mesorhizobium* strains, cultivars, and NPSB fertilizer (**Table 2**). The local variety planted without inoculation and fertilizer application had the shortest plant height (38.05 cm), while the Arerti variety with the CP-M41 strain and blended NPSB fertilizer application had the longest plant height (56.4 cm). Finally, the local variety with the CP-EAL 029 strain and NPSB fertilizer application came in second place with 49.73 cm (**Table 5**). When compared to an unfertilized and uninoculated treatment, the interaction of the NPSB * CP-M41 strain produced the longest plant height. This could be attributed to the strain’s improved ability to fix nitrogen, which increased plant uptake and potentially improved chickpea vegetative growth (Admasu, 2019). It could possibly be because S boosted the crop’s availability of nutrients (N, P, and B) and the activity of the rhizobia and meristematic tissue (Singh et al., 2018). The greatest value may be the result of S activating certain enzymes that cause cell elongation and division, which are the main causes of the CP-M41 *Rhizobium* strain’s rise in plant height. The enhanced availability of nitrogen in the soil for plant roots to absorb may have sufficiently enhanced vegetative growth by increasing cell division and elongation, which could account for the notable increase in plant height in response to the combined application of NPSB with *Rhizobium* inoculation. An increase in plant height with the combined effect of *Rhizobium* inoculation and inorganic fertilizer on chickpeas has also been well-documented by (Singh et al., 2018; Admasu, 2019).

#### 3.4.2 Number of main and secondary branches

*Mesorhizobium* strains, cultivars, and mixed NPSB fertilizer applications all had a substantial (P<0.001) impact on the number of primary or main and secondary branches per chickpea plant (**Table 2**). The local variety inoculated with the CP-EAL 029 strain under NPSB fertilizer application (3.5 branches) obtained a lower number of main branches per plant (3.7) than the Arerti variety inoculated with the CP-M41 strain and supplied with 121 kg NPSB ha^-1^.

**Figure 4:**
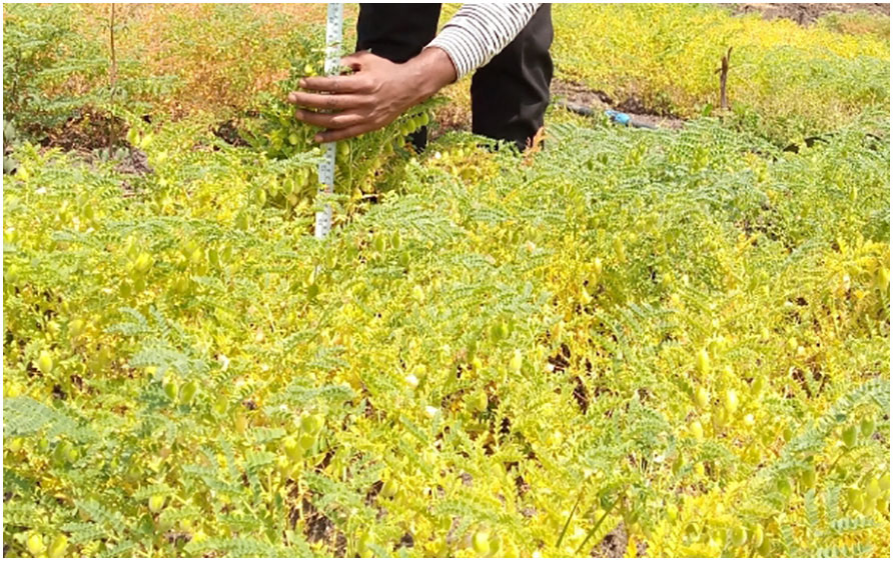
Showing the growth performance of inoculated Arerti chickpea plants in the experimental field.

Similarly, the Arerti variety that was inoculated with the CP-M41 strain and given 121 kg NPSB ha^-1^ had the highest number of secondary branches per plant (14.23). Conversely, the local chickpea varieties without NPSB application and the uninoculated Arerti variety had the lowest secondary branch per plant scores of 6.63 and 2.06, respectively (**Table 5**). **Figure 4** shows the high-growth performance of inoculated Arerti chickpea plants in the experimental field.

The rhizobia inoculant may have enhanced the ratio of N and improved P uptake because of the presence of S, which could account for the increased number of branches caused by the inoculant with NPSB fertilizer application (Raj et al., 2017). This may be because P and S work in concert to help plants use large amounts of nutrients through the development of their nodules and well-developed root systems, which may have improved vegetative growth (Kumar et al., 2017). The greatest value could indicate that P, S, and B were involved in the growth regulator and chlorophyll synthesis process, particularly in cell division, which may have resulted in more branching. In line with this result (Uddin et al., 2014), noticed that applying various amounts of P and S along with the inoculant greatly enhanced the number of branches in chickpeas. Similarly (Kiros and Atsede, 2020), discussed how the growth characteristics of chickpea crops in Ethiopia’s Tigray Region were significantly impacted by the simultaneous application of NPSB fertilizer and *Rhizobium* inoculation.

#### 3.4.3 Dry weight of shoot per chickpea plant

The dry weight of shoot per chickpea plant was significantly (P¾0.001) influenced by the three-way combined effects of *Mesorhizobium* strains, cultivars, and NPSB fertilizer (**Table 2**). The result revealed that the maximum shoot dry weight per plant of 48.32 g was obtained from the Arerti variety when planted with the combined application of CP-M41 strain and NPSB blend fertilizer, whereas the lowest value of 29.89 g was obtained from the local cultivar at un-inoculated and unfertilized treatment (**Table 5**). Genetic variations in the varieties’ responses to both inputs may account for the notable range in shoot dry matter output observed between them after applying NPSB and inoculating with various *Mesorhizobium* inoculants. Chickpea shoots dry weight increased when NPSB fertilizer and *Mesorhizobium* inoculant were applied together. This was primarily because P in the blended NPSB fertilizer led to a well-developed root system with a higher capacity to fix nitrogen, S improved nitrogenase activity and N fixation, and the inoculant increased the number of rhizobia and improved N fixation, which improved N availability and improved plant growth and development (Singh et al., 2018). Because of the unique activities of S, P, and B as well as the CP-M41 strain, which enhanced metabolic activity and nutrient utilization and led to the highest vegetative growth and accumulation of dry matter, the highest value may also be the result of the biggest leaf surface area. The application of S and B in NPSB and the CP-M41 *Rhizobium* strain may have contributed to the highest value by enhancing N and P uptake, stimulating photosynthetic activity, and the synthesis of chloroplast protein, all of which led to a larger output of dry matter. Singh et al. (2018) found that the application of rhizobia and nutrients together raised the shoot dry weight of chickpeas compared to the unfertilized and uninoculated control, which is consistent with this outcome.

#### 3.4.4 Dry weight of root per chickpea plant

**Figure 5:**
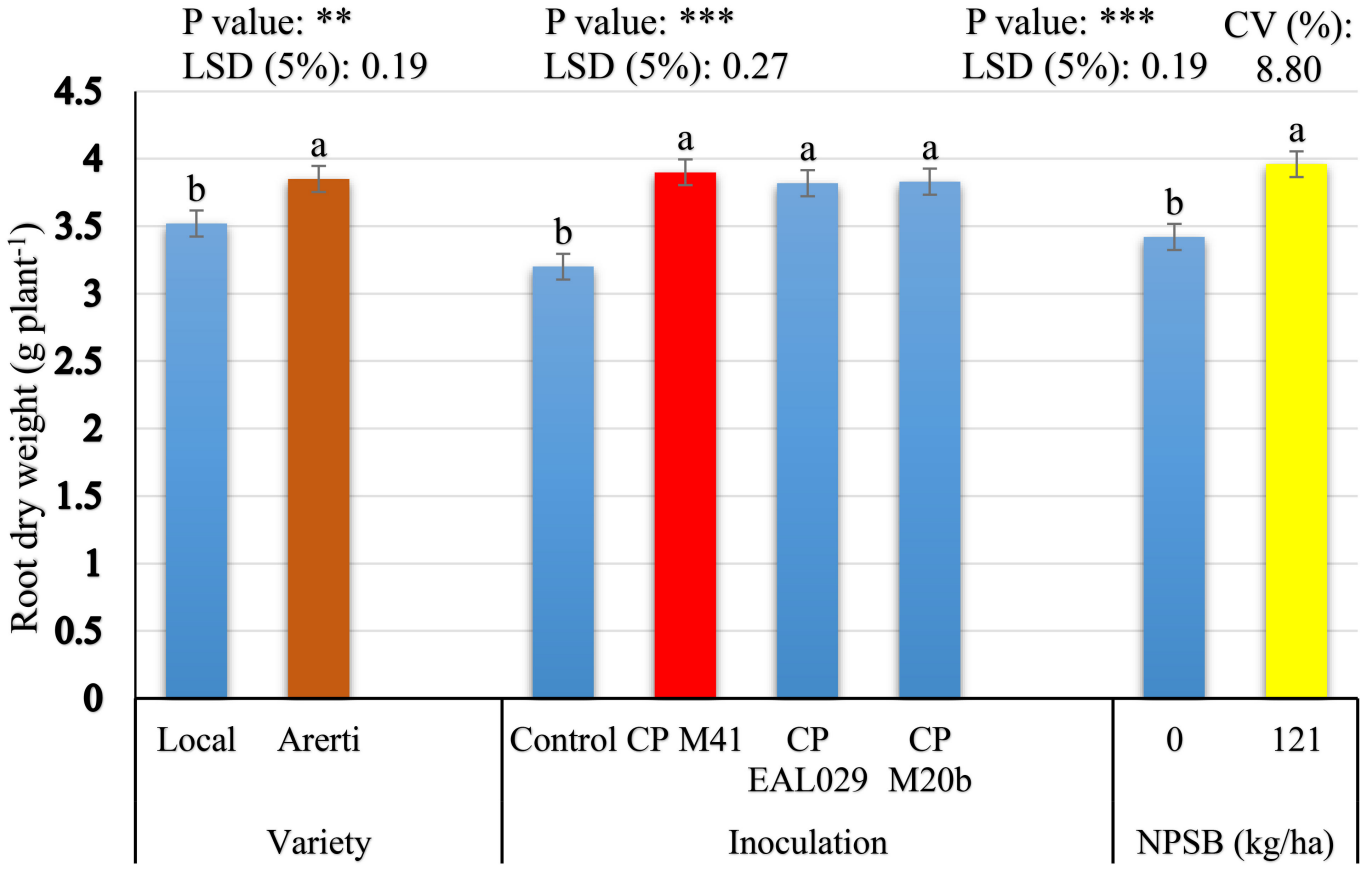
Root dry weight of chickpea as influenced by Varieties, *Mesorhizobium* strains, and NPSB fertilizer application. The main effect means of each factor within the bar graph followed by the same letter (s) are not significantly different from each other at a 5% level of significance based on the Fishers LSD test; ** = significant at P ≤ 0.01; *** = significant at P ≤ 0.001; LSD, least significance difference; CV, Coefficient of variation.

The principal effects of varieties, *Mesorhizobium* strains, and NPSB application significantly influenced root dry weight per plant of chickpea. However, their two-way and three-way interaction effects were not significant for this parameter (**Table 2**). Variety Arerti scored significantly (P ¾ 0.01) the highest mean root dry weight per chickpea plant (3.86 g) than the Local variety (3.52 g) (**Figure 5**). This significant variation might be because of cultivars’ genetic differences. It has been shown that there is a notable variation in the root dry weight per plant throughout chickpea cultivars (Molla, 2016). Similarly, *Mesorhizobium* inoculation treatments significantly (P ≤ 0.001) affected the dry weight of root per chickpea plant. The chickpea treated with CP M41 had the largest root dry weight per plant (3.90 g), followed by CP M20b (3.83 g). Conversely, uninoculated plants had the lowest dry weight of roots per plant (3.20 g) (**Figure 5**). After inoculating chickpeas with the CP M41 strain, the dry weight of the roots rose by 17.95% compared to the uninoculated plants. This study showed that when compared to the uninoculated group, inoculation greatly increased root dry weight per plant. The increased root dry weight per plant that the inoculant produced may have been the consequence of greater root nodulation, which boosted soil N availability and promoted plant growth and development (Admasu, 2019). Moreover, the highest root dry weight from CP M41 might be due to its higher N fixing capacity (Wondwosen et al., 2016). Similarly (Tamiru and Girma, 2019b), also found that the root dry weight of chickpeas rose after being injected with rhizobial inoculants.

Regarding the NPSB fertilizer treatments, the result in **Figure 5** indicates that NPSB fertilizer treatments significantly (P ≤ 0.001) increased the root dry weight per plant by 13.64% over unfertilized treatment. The application of NPSB fertilizer may have raised the root dry weight because P is necessary for root growth and development (Admasu, 2019). It is generally known that P causes nodule development and affects the *rhizobium*-legume symbiosis’s effectiveness, which increases nitrogen fixation and, in turn, root biomass (Singh et al., 2018). The highest root dry weight value seen in chickpeas treated with blended NPSB fertilizer may also be attributable to the S included in NPSB, which increased the uptake of N and P, sparked photosynthetic activity, and encouraged the synthesis of chloroplast protein, all of which increased the production of root dry matter (Merkebu, 2019). Consistent with this outcome (Merkebu, 2019), found that the application of NPSB fertilizer increased the root dry weight of the Mung bean crop by 41.46% over the control treatment.

### 3.5 Effects of *Mesorhizobium* strains, varieties, and NPSB fertilizer on yield components of chickpea

#### 3.5.1 Total pod count for each plant

The pod number per chickpea plant was significantly affected by the three-way (p¾0.01) interaction of cultivars, inoculants, and NPSB fertilization (**Table 6**). The maximum pod number per plant (84.6) was obtained from CP-M41 inoculated Arerti variety under NPSB fertilizer application. In contrast, the lowest pod number per chickpea plant (54.41) was obtained from the Local variety planted without inoculation and NPSB fertilizer application (**Table 7**). Arerti variety inoculated with CP-M41 with NPSB application scored almost 8% higher pod number than the local variety received the same inoculant and fertilizer. This might be attributed to the relatively higher nutrient uptake and/or utilization efficiency of the Arerti variety than the local variety.

**Table 6:** Mean squares ANOVA for yield components of chickpea to seed inoculation with *Mesorhizobium* strains, Varieties, and NPSB fertilization.

SV	DF	NPPL (no)	NSP (no)	HSW (g)
**Rep**	2	2.33	0.0005	5.808
**V**	1	201.31***	0.0085***	72.201***
**RI**	3	366.20***	0.0081***	72.977***
**NPSB**	1	746.55***	0.026***	121.444***
V*RI	3	142.60***	0.0055***	8.494***
V*NPSB	1	35.70*	0.00083*	1.573**
RI*NPSB	3	40.30**	0.00096***	0.641*
V*RI*NPSB	3	32.66**	0.00165***	0.524*
**Error**	30	5.97	0.00012	0.173
**Total**	47			

SV= Sources of variation; * Significant at p ≤ 0.05; ** significant at p ≤ 0.01; *** significant at p ≤ 0.001 by LSD; NS, non- significant at p > 0.05; DF, Degree of freedom; N, nitrogen; P, phosphorus; S, Sulfur; B, boron; V, Variety; RI, *Mesorhizobium* inoculants; NPPL, number pods per plant; NSP, number of seeds per pod; HSW, Hundred seed weight; GY, Grain yield; BY, Biomass yield; HI, Grain harvest index.

**Table 7:** Three-way interaction effects of varieties, *Mesorhizobium* strains, and NPSB fertilizer on yield components and yield traits of chickpea in Cheha district.

Treatments			NP	NSP	HSW (g)	BY (kg)	GY (kg)	SY (kg)	HI (ratio)
Variety	NPSB (kg ha^-1^)	Strains							
Arerti	Control	Control	58.7^ij^	1.06^hi^	21.3^h^	4290.4^f^	1971.9^h^	2318.5^efghi^	0.46^g^
		CP M41	66.9^ef^	1.10^def^	26.6^c^	5073.1^cd^	2669.9^d^	2403.2^def^	0.52^cd^
		CP EAL-029	63.3^fgh^	1.08^gh^	25.1^def^	4914.0^d^	2523.7^e^	2390.4^defg^	0.51^e^
		CP M20b	64.8^efgh^	1.09^fg^	25.5^cde^	4961.8^d^	2580.9^e^	2380.9^defgh^	0.52^cd^
	121	Control	61.8^hi^	1.09^fg^	24.2^fg^	4901.4^d^	2412.7^f^	2488.7^bcd^	0.49^f^
		CP M41	84.6^a^	1.21^a^	31.1^a^	5924.3^a^	3177.2^a^	2747.2^a^	0.55^a^
		CP EAL-029	71.5^cd^	1.12^cd^	28.2^b^	5233.2^c^	2816.6^c^	2416.1^cde^	0.53^b^
		CP M20b	74.2^bc^	1.13^bc^	29.1^b^	5442.7^b^	2890.7^bc^	2552.0^b^	0.53^b^
Local	Control	Control	54.4^k^	1.03^j^	19.2^i^	3726.8^g^	1693.9^i^	2032.9^k^	0.45^h^
		CP M41	60.8^hij^	1.06^hi^	23.0^g^	4615.1^e^	2405.9^f^	2209.2^ij^	0.52^cd^
		CP EAL-029	66.5^efg^	1.09^efg^	24.6^ef^	4905.8^d^	2620.4^d^	2285.4^ghij^	0.53^b^
		CP M20b	62.5^ghi^	1.07^ghi^	23.3^g^	4655.7^e^	2427.8^f^	2227.8^ij^	0.52^cd^
	121	Control	57.2^jk^	1.06^i^	21.3^h^	4264.7^f^	2115.7^g^	2149.0^jk^	0.49^f^
		CP M41	65.9^efg^	1.09^efg^	25.8^cde^	5130.4^c^	2720.1^d^	2410.4^hij^	0.53^b^
		CP EAL-029	77.4^b^	1.15^b^	28.0^b^	5486.7^b^	2952.2^b^	2534.5^bc^	0.53^b^
		CP M20b	68.3^de^	1.11^de^	26.3^cd^	5150.5^c^	2727.4^d^	2423.1^fghi^	0.53^b^
P value			**	***	*	**	**	**	**
CV (%)			3.62	1.12	2.88	2.25	1.93	3.22	1.36

Interaction means within a column followed by the same letter (s) are not significantly different from each other at a 5% level of significance based on the Fishers LSD test; * = significant at P ≤ 0.05; ** = significant at P ≤ 0.01; *** = significant at P ≤ 0.001; CV, Coefficient of variation; NP, number of pods; NSP, number of seeds per pod; HSW, hundred seed weight; BY, Biomass yield; GY, Grain yield; SY, Straw yield; HI, Harvest index.

Furthermore, the maximum number of pods per plant from the combined effects of NPSB * CP-M41 on Arerti may be the result of S’s critical function in plant growth and development, which includes promoting the production of nodules, enzyme activation, and chlorophyll and nitrogenase (Beza, 2017), and because the CP-M41 strain performs better at fixing N than the other strains.

It could also be because there was enough N available through BNF and P, which enhanced the growth of main and secondary branches as well as plant height, all of which could have led to the production of more pods overall. The positive association between plant height and pod quantity, as well as the principal and secondary branches, may lend support to this. The benefits of N and P on chickpea pod production may be linked to their ability to support both vegetative and reproductive processes, which enhances photosynthetic efficiency and improves the partitioning of carbohydrates, both of which increase the number of pods per plant (Kumar et al., 2017; Admasu, 2019). The positive effects of inoculants may be due to a greater amount of nitrogen produced through nitrogen fixation, which promotes vegetative growth and plant height, thus increasing the number of pods per plant. This in turn could be attributed to the availability of P, S, and B, which would increase the intensity of photosynthesis, nitrogen fixation, root development, flowering, seed formation, and fruiting. The higher number of pods in plant 1 of strain NPSB*CP-M41 may be due to the combination of the positive effect or B role on stamen and pollen formation, which may increase the number of pods produced in the plant. S plays many important roles in plant growth and development, including chlorophyll formation and nitridation, P promotes nodulation and enzyme activation, and the nitrogen fixation efficiency of strain CP-M41 was higher than that of strains CP-EAL 029 and CP-M20b. Similarly (Kiros and Atsede, 2020), also showed that, in comparison to the control, the combined application of NPSB fertilizer and *Rhizobium* inoculation enhanced the number of pods per plant.

#### 3.5.2 Quantity of seeds in each pod

The three-way (p<0.001) interaction effect of cultivars, NPSB fertilizer application, and inoculation with *Mesorhizobium* strains affected the number of seeds per pod of chickpea substantially (**Table 6**). **Table 7** illustrates that the variety Arerti exhibited the highest number of seeds per pod (1.213) when NPSB fertilization (121 kg ha^-1^) was combined with the CP-M41 strain. Conversely, the local variety, which was planted without inoculation and NPSB fertilizer application, had the lowest number of seeds per pod (1.03). The interaction of NPSB blended fertilizers with CP-M41 inoculant resulted in a 12.61% increase in the number of seeds per pod of the Arerti chickpea variety as compared to the control. Improved nodule growth, protein synthesis, fruit, and seed formation—all of which may lead to more seeds—may be the consequence of an appropriate supply of N, P, S, and B nutrients from NPSB fertilizer mixed with inoculant (Singh et al., 2018; Nuru, 2020). The photosynthetic function of N, the seed formation capacity of P, the metabolic capacity of S and B, and the nitrogen fixation ability of *Rhizobium* strain CP-M41 may have contributed to the greater seed number Pod^-1^ in this study. The result of the present study conformed with (Endalkachew et al., 2018; Admasu, 2019) who observed a considerable increase in the number of grains per pod in chickpeas when P-containing fertilizer and inoculant were applied together.

#### 3.5.3 100-seed weight (HSW)

Data analysis revealed that *Mesorhizobium* strains, NPSB fertilizer, and cultivars had a substantial (P<0.05) three-way interaction effect on chickpea HSW (**Table 6**). The variety Arerti at the combined application of NPSB (121 kg ha^-1^) with the CP-M41 strain had the highest hundred-grain weight (31.14 g), whereas the local variety at the control treatment had the lowest (19.16 g). The HSW of the Arerti variety was greatly raised by *Mesorhizobium* inoculation with CP-M41 strain and NPSB fertilizer treatment, as compared to the Arerti variety and local variety cultivated without inoculation and fertilization, respectively, by 31.66% and 38.47% (**Table 7**).

The highest grain weight observed after applying NPSB fertilizer and inoculant together may have resulted from improved crop plant growth and development (vegetative and reproductive), which was made possible by increased availabilities of N, P, S, and B from fertilization and inoculation. This increased supply of assimilates (dry matter) to grain led to its eventual weight gain (Wazir et al., 2018). The highest result from the combination of NPSB *CP-M41 may be the result of P and S administration, which is essential for crop growth and involves protein synthesis, respiration, photosynthesis, and nitrogen metabolism. The weight of the seed can be significantly increased by the translocation of the assimilated photosynthates from the vegetative plant parts to the seed. Additionally, improved crop plant development and growth brought about by S supply and N uptake may have enhanced the amount of assimilates available for seed, which ultimately gained more weight. The rise in 100-grain weight demonstrated that micronutrients (B) and *Rhizobium* inoculation (CP-M41) are required for stronger, healthier chickpea seeds. Similarly (Kiros and Atsede, 2020), showed that the hundred-seed weight of chickpeas rose when rhizobia inoculant and NPSB fertilizer were applied together. Other researchers (Uddin et al., 2014; Mulugeta et al., 2018; Wazir et al., 2018), have found that adding inorganic fertilizer and inoculant at the same time enhanced chickpea grain weight. Another study (Beza, 2017), said that the impact of using inorganic fertilizers along with *Rhizobium* inoculation on the 100-seed weight of chickpeas is noteworthy.

### 3.6 Effects of *Mesorhizobium* Strains, Varieties, and NPSB Fertilizer on Yield Traits of Chickpea

#### 3.6.1 Aboveground dry biomass yield

The biological yield of a crop determines its productivity in considerable part. Large-scale biomass production is one of the characteristics of seed yield. One of the requirements for crop growth is an increase in the buildup of dry matter. The total above-ground dry biomass yield of the chickpea crop was significantly (P<0.01) impacted by the three-way interaction effect of cultivars, NPSB fertilizer treatment, and *Mesorhizobium* strains inoculation (**Table 8**). Variety Arerti produced the maximum total above-ground dry biomass (5924.33 kg ha^-1^) when NPSB fertilization was combined with the CP-M41 *Mesorhizobium* strain. Conversely, the local variety planted without inoculation or fertilizer application yielded the lowest total above-ground dry biomass production (3726.83 kg ha^-1^) (**Table 7**). When compared to the local variety produced without inoculation and NPSB fertilization, the Arerti variety grown under the combined application of CP M41 strain and blended NPSB fertilizer improved the above-ground biomass output of chickpea by 37.09%.

**Table 8:** Mean squares ANOVA for yield traits of chickpea to seed inoculation with *Mesorhizobium* strains, Varieties, and NPSB fertilization.

SV	DF	GY (kg)	BY (kg)	SY (kg)	HI (Ratio)
**Rep**	2	2851.833	1576.304	3721.66	0.000152
**V**	1	368638.380***	1845536.333***	564525.63***	0.000675***
**RI**	3	1323539.286***	1971354.516***	65756.8***	0.010905***
**NPSB**	1	1459588.501***	2965798.041***	264211.36***	0.012675***
V*RI	3	156850.604***	440252.817***	71934.62***	0.001347***
V*NPSB	1	18198.999**	56189.242*	10432.38^NS^	0.000300*
RI*NPSB	3	15820.094**	27695.382^NS^	3831.19^NS^	0.000113^NS^
V*RI*NPSB	3	8652.700**	72339.991**	36476.69**	0.000150**
**Error**	30	2403.912	12897.06	5914.39	0.000045
**Total**	47				

SV, Sources of variation; * Significant at p ≤ 0.05; ** significant at p ≤ 0.01; *** significant at p ≤ 0.001 by LSD; NS, non- significant at p > 0.05; DF, Degree of freedom; N, nitrogen; P, phosphorus; S, Sulfur; B, boron; V, Variety; RI, Mesorhizobium inoculants; NPPL, number pods per plant; NSP, number of seeds per pod; NSPL, number of seeds per plant; HSW, Hundred seed weight; GY, Grain yield; BY, Biomass yield; HI, Grain harvest index.

The combined application of inorganic fertilizers and CP-M41 rhizobia inoculant significantly increased nodulation and improved plant vegetative growth and development, which leads to increased dry matter yield compared to the single use of inorganic fertilizer or Rhizobia strain. This could account for the increased total dry biomass yield caused by the interaction of the CP-M41 strain with NPSB fertilizer application (Endalkachew et al., 2018). The observed increases in the total dry biomass yield are consistent with those seen for growth-related variables including plant height, branch count, and shoot dry weight, and they may also be the result of increased N, P, S, and B availability. Nonetheless, a decreased overall biomass output may have been the consequence of the CP-M20b and CP-EAL 029 strains performing worse than the CP-M41 strain. The use of inorganic fertilizer and CP-M41 strain may have enhanced the availability of N and other nutrients P, S, and B, leading to a notable increase in the crop’s vegetative development. This, in turn, may have contributed to the rise in the biomass yield of common beans. This suggests that in terms of promoting vegetative development, the recently applied Rhizobia strain is more successful than the native *Rhizobia* strain. Admasu (2019) reported comparable outcomes.

#### 3.6.2 Grain yield

Plant systems undergo a variety of physiological, biochemical, phonological, and morphological processes that result in the creation of dry matter and its conversion into economic yield. A variety’s seed output is the product of the interaction between its genetic composition and the growing environment (Tamiru et al., 2021). The three-way relationship between cultivars, *Mesorhizobium* strains, and NPSB fertilizer treatment affected chickpea grain output in a significant (P<0.01) way (**Table 8**). The results of the interaction showed that the Arerti variety produced the highest grain yield (3177.16 kg ha^-1^) when the CP-M41 strain and NPSB fertilizer were applied together (121 kg ha^-1^). This was followed by the use of the Local variety when CP-EAL 029 strain and NPSB fertilizer were applied together (2952.19 kg ha^-1^), and the Local variety produced the lowest grain yield (1693.91 kg ha^-1^) when it was grown without inoculation and fertilizer application (**Table 7**). The outcome demonstrated that, in comparison to cultivating local varieties without inoculation and fertilization, planting the Arerti variety with CP M41 strain and NPSB combined application enhanced the grain production of chickpeas by 46.68%. The results also indicated that the Arerti variety’s grain yield increased by 26.14% and 18.27% over the control check, respectively, following *Mesorhizobium* inoculation with the CP-M41 strain and NPSB fertilizer application. Additionally, the interaction between the CP-M41 strain and NPSB fertilizer produced a 37.93% increase in grain yield.

The critical role that N, P, S, and B nutrients play when combined with the CP-M41 strain to improve plant growth, biomass production, nodule formation and development, N_2_ fixation, and yield contributing characteristics may account for the highest grain yield result as a result of the interaction effects of the strain and NPSB blend fertilizer application. This may have happened as a result of P, S, and B’s beneficial impacts on the nitrogen fixation process, where a higher N supply from inoculation led to improved plant growth, which in turn produced a better yield (Beza, 2017; Singh et al., 2018). This higher result in terms of grain yield could be due to the positive role of the combination of strains N, P, S, B, and CP-M41. Nitrogen in the early growth stages promoted vegetative growth and create conditions for high yield, played a key role in the formation of chlorophyll and proteins, and directly increased the protein content of the plant and thus performance (Endalkachew et al., 2018). P increased cellular respiration in starch, protein, and lipid metabolism plays an essential role in metabolic and energy production reactions, builds phospholipids and nucleic acids, and also stimulates flowering and seed formation, which can increase yields (Endalkachew et al., 2018). S increased chlorophyll concentration, root nodules, and dry matter production, which contributed to yield increase (Beza, 2017). B is involved in nodule development and supports the utilization of P and N (Bolanos et al., 1996). This has a significant impact on the yield components and leads to an increase in yield (Singh et al., 2018). The *Rhizobium* strain also increased the formation of more nodules and improved nitrogen fixation in the plant atmosphere, resulting in higher yields (Endalkachew et al., 2018).

But, in CP-M20b *NPSB and CP-EAL 029 * NPSB interactions, the lower performance of both CP-M20b and CP-EAL 029 strains than the CP-M41 strain might have resulted in significantly lower grain yield values than CP-M41 * NPSB strain interaction. This outcome is consistent with (Mulugeta et al., 2018) who found that using P and *Mesorhizobium* inoculant together greatly enhanced chickpea grain production (Endalkachew et al., 2018). also found that, when rhizobia inoculant and P were applied together to chickpea plants, grain production increased considerably. Similarly (Kiros and Atsede, 2020), showed that applying *Rhizobium* inoculants and NPSB fertilizer together had a synergistic effect on chickpea production, increasing it by 34% over the untreated control (Das et al., 2016). have also seen a rise in chickpea grain production when P and S are applied together.

#### 3.6.3 Straw yield

*Mesorhizobium* strains, NPSB treatment, and variety all had a three-way interaction that significantly (P<0.01) affected chickpea straw yield (**Table 8**). The local variety had the lowest straw production (2032.91 kg ha^-1^) when planted without inoculation and fertilizer application, while the Arerti variety injected with the CP M41 strain and applied NPSB fertilizer yielded the maximum yield (2747.16 kg ha^-1^). The combined use of the CP-M41 strain with NPSB fertilizer increased the straw yield of the Arerti variety by 25.99% over that of the local variety grown under the control plot (**Table 7**). The outcome showed that, in comparison to the single application of NPSB or inoculant, the combined application of rhizobia inoculant and NPSB to legume plants greatly enhanced nodulation and enhanced vegetative growth and development of plants, which resulted in greater straw yield (Beza, 2017; Endalkachew et al., 2018; Tamiru et al., 2021). The enhanced availability of N, P, S, and B is what is responsible for the reported improvements in straw production; these improvements are consistent with those noted for growth-related parameters including plant height, number of branches, and shoot dry weight.

In chickpeas, the administration of P, S, and B in combination with the inoculant increased plant height, branch count, nodule count, dry weight of nodules, and straw production (Admasu, 2019). Similarly (Wazir et al., 2018), furthermore noted enhanced straw yield when P fertilizer and inoculant were applied simultaneously to chickpea plants. In addition (Fatima et al., 2007), found that the yield of cowpea straw rose by 63% when P and inoculation were applied together.

#### 3.6.4 Harvest index

As shown in **Table 8**, *Mesorhizobium* strains, NPSB application, and variety all had a three-way interaction impact that was statistically significant (P<0.01) on the chickpea harvest index (HI). Notably, variety Arerti showed the highest harvest index (0.55) when cultivated with CP-M41 inoculant and NPSB fertilizer application, while variety Local showed the lowest HI (0.45) when grown without inoculant and fertilizer. The Arerti chickpea variety grown with the combined application of CP M41 strain and blended NPSB fertilizer grew at a higher yield index (18.18%) than the local variety cultivated without inoculation or fertilization.

A possible explanation for the increase in harvest index caused by the NPSB fertilizer and *Rhizobial* inoculant is that more photosynthetic energy is produced, which eventually partitions to the grains as opposed to the straw (Admasu, 2019). Additionally, the application of balanced NPSB nutrients raised the hundred grains weight of the chickpea crop, and inoculation increased the number of effective nodules per plant and N availability, which in turn enhanced dry matter partitioning in favor of grain showing a larger harvest index (Endalkachew et al., 2018; Nuru, 2020). The results of earlier studies also suggested that *Mesorhizobium* inoculation and the administration of N, P, S, and B nutrients were beneficial in raising the harvest index of chickpea crops (Beza, 2017).

### 3.7 Correlation analysis

The nodulation traits, growth parameters, yield components, and other yield traits of chickpeas strongly and very highly significantly positively associated with grain yield of chickpea, and therefore contributed to the maximum yield of the crop, according to the correlation analysis of nodulation, growth, yield components, and yields of chickpeas (**Table 9**).

**Table 9:** Relationship among nodulation, growth, grain yield, and yield traits of chickpea crop.

	TNN	ENN	NV	NDW	SHDW	PHPL	NPB	NSB	NPPL	NSP	DBY	SY	HSW	HI	GY
**TNN**	1														
**ENN**	0.93^***^	1													
**NV**	0.91^***^	0.94^***^	1												
**NDW**	0.92^***^	0.95^***^	0.94^***^	1											
**SHDW**	0.91^***^	0.9^***^	0.95^***^	0.93^***^	1										
**PHPL**	0.76^***^	0.76^***^	0.83^***^	0.76^***^	0.8^***^	1									
**NPB**	0.8^***^	0.82^***^	0.92^***^	0.82^***^	0.85^***^	0.79^***^	1								
**NSB**	0.88^***^	0.92^***^	0.91^***^	0.94^***^	0.9^***^	0.74^***^	0.81^***^	1							
**NPPL**	0.85^***^	0.9^***^	0.93^***^	0.91^***^	0.91^***^	0.84^***^	0.87^***^	0.9^***^	1						
**NSP**	0.89^***^	0.93^***^	0.91^***^	0.94^***^	0.88^***^	0.81^***^	0.8^***^	0.89^***^	0.91^***^	1					
**DBY**	0.94^***^	0.9^***^	0.94^***^	0.91^***^	0.93^***^	0.8^***^	0.87^***^	0.89^***^	0.9^***^	0.9^***^	1				
**SY**	0.83^***^	0.78^***^	0.78^***^	0.79^***^	0.82^***^	0.49^***^	0.72^***^	0.71^***^	0.63^***^	0.77^***^	0.88^***^	1			
**HSW**	0.9^***^	0.89^***^	0.93^***^	0.92^***^	0.92^***^	0.74^***^	0.86^***^	0.92^***^	0.9^***^	0.89^***^	0.94^***^	0.79^***^	1		
**HI**	0.89^***^	0.9^***^	0.93^***^	0.9^***^	0.85^***^	0.75^***^	0.91^***^	0.91^***^	0.87^***^	0.84^***^	0.9^***^	0.64^***^	0.91^***^	1	
**GY**	0.91^***^	0.89^***^	0.94^***^	0.89^***^	0.91^***^	0.77^***^	0.92^***^	0.89^***^	0.89^***^	0.86^***^	0.97^***^	0.75^***^	0.93^***^	0.95^***^	1

TNN, Total nodule number; ENN, Effective nodules number; NV, Nodule volume; NDW, Nodule dry weight; SHDW, Shoot dry weight; PHPL, Plant height per plant; NPB, Number of the primary branch; NSB, Number of secondary branch; NPPL, Number of pods per plant; NSP, Number of seeds per pod; DBY, Dry biomass yield; SY, Straw yield; HSW, Hundred seed weight; HI, Harvest index; GY, Grain yield. *** = Very highly significant correlation.

### 3.8 Economic Analysis of Treatment Effects

Eleven (11) of the sixteen (16) treatments were dominated and five were non-dominated, as per the dominance analysis (**Table 10**). The treatment with the Arerti variant at NPSB and inoculants application showed the largest variable cost (5930.6 birr), while the untreated control treatment showed the lowest variable cost (2,500.00 birr). All other treatments fell in between these two treatment categories (**Table 10**). The prevalent therapies are excluded from additional economic analysis due to their high total cost of variable but lower net benefit. However, the other treatments were not dominant and were taken into account for the marginal rate of return because they had higher variable costs and net benefits. Based on the data from the economic analysis, all marginal rates were higher than 100%, falling within an acceptable range (CIMMYT, 1988). According to (CIMMYT, 1988), treatments with high net benefit, reasonably low variable cost with an acceptable and maximal MRR become the tentative economically optimal treatments. Consequently, even though the Arerti variety inoculated with the same strain and the Local variety with CP-M41 *Mesorhizobium* inoculation ranked first and second, respectively, among the treatments with the highest marginal rate of return in percent, the benefit realized was less than that of the treatment involving the combined use of the Arerti variety with CP-M41 *Mesorhizobium* inoculant and NPSB application.

**Table 10:** Dominance analysis of treatments and marginal analysis of un-dominated treatments.

Treatments	TVC (ETB ha^-1^)	NB (ETB ha^-1^)	Dominance	MC (ETB ha^-1^)	MB (ETB ha^-1^)	MRR (%)
L	2500	40694.6	UD	–	–	–
L x S1	2830	58519.7	UD	330	17825.1	5401
L x S2	2830	65342	UD	0	6822.3	–
L x S3	2830	59080.1	D			
A	3200	63843.2	D			
A x S1	3530	87245.6	UD	700	21903.6	3129
A x S2	3530	82275.2	D			
A x S3	3530	84221.2	D			
L x NPSB	4900.6	49048.7	D			
L x S1 x NPSB	5230.6	62983.1	D			
L x S2 x NPSB	5230.6	70050.2	D			
L x S3 x NPSB	5230.6	63553.6	D			
A x NPSB	5600.6	76431.8	D			
A x S1 x NPSB	5930.6	102092.6	UD	2400.6	14847	618
A x S2 x NPSB	5930.6	89834.2	D			
A x S3 x NPSB	5930.6	92352.2	D			

UD, Un-dominated; D, Dominated treatments; TVC, total variable cost, NB, net benefit (GB-TVC); MC, Marginal cost (Change in total variable cost between treatments); MB, Marginal benefit (Change in net benefits between treatments); MRR, Marginal rate of return (MB/MC * 100).

Thus, according to the budget summary of economic analysis, the Arerti variety planted with CP-M41 *Mesorhizobium* strain and 121 kg NPSB ha^-1^ application yielded the maximum net return (102092.6 ETB ha^-1^) with an appropriate marginal rate of return (618%). This suggests that by altering current practices and implementing the new treatment, growers in the study area can gain an additional 6.18 ETB/ha for every 1 ETB expense. Arerti variety planted without NPSB fertilizer and with *Mesorhizobium* CP-M41 inoculation came in second with a calculated net return of 87245.6 ETB ha^-1^, while local variety planted without inoculation and NPSB fertilizer application had the lowest net economic return (40694.6) (**Table 6**). In the Cheha area, Gurage zone, SNNPRS of Ethiopia, it was determined that the Arerti variety, *Mesorhizobium* inoculation with strain CP-M41, and 121 kg NPSB ha^-1^ treatment were economically feasible. In line with this finding (Kiros and Atsede, 2020), revealed that at the Hatsebo research site in the Laelay maichew district, Tigray regional state, Northern Ethiopia, planting the cultivar Arerti with blended NPSB application and Rhizobia inoculation produced the highest net benefit (67132.2025 ETB ha^-1^) with an acceptable marginal rate of return (4106.48%) compared to other treatments.

## 4 Conclusion and recommendations

A field experiment was carried out in Cheha district of Gurage zone in Southern Ethiopia to assess the effects of four levels of *Mesorhizobium* strains (un-inoculated control, inoculated with strains CP-M41, CP EAL-029, and CP-M20b) and two levels of blended NPSB fertilizer (0 and 121 kg ha^-1^) on the growth, nodulation, yield, and yield components of two chickpea varieties (Arerti and local varieties). Although nitrogen fixation, growth, and productivity of chickpeas are influenced by nitrogen, phosphorus, sulfur, boron, and Rhizobia bio-fertilizers (inoculants), the majority of farmers in the study area did not apply any fertilizer materials to increase crop productivity, leading to productivity issues. The present study showed that the number of total nodules, effective nodule number, volume of nodules, dry weight of nodules, plant height, number of primary and secondary branches, shoot dry weight, number of pods, number of seeds per pod, hundred seed weight, above ground dry biomass yield, grain yield, and straw yield of the chickpea crop were all significantly impacted by the three-way interaction effect of varieties, *Mesorhizobium* inoculants, and NPSB fertilizer.

From the results of the research carried out, it can be seen that the most important values ​​of most of the recorded agronomic parameters such as the total number of nodules, the actual number of nodules, the volume of nodules, the dry weight of nodules, the height of the plant, the number of primary - and secondary branches and the dry weight of the shoots., number of pods, number of seeds per pod, weight of hundreds of seeds, aboveground dry biomass yield, grain yield, straw yield, and chickpea crop index were recorded based on the interaction of NPSB compound fertilizer with strain *Mesorhizobium CP*-M41 for chickpea cultivar Arerti. In addition, the study also showed that the maximum economic gain with Arerti variety was obtained by inoculating CP-M41 *Mesorhizobium* and 121 kg NPSB ha^-1^. The results also showed that for both chickpea varieties, the combined application of *Mesorhizobium* inoculant and NPSB fertilizer increased production more than a single application of compound fertilizer and NPSB inoculant.

Therefore, for better production and profitability of chickpeas in the Southern part of Ethiopia and areas similar to the study area, it is recommended that producers shall better use NPSB blend fertilizer in combination with the CP-M41 strain along with the improved Arerti chickpea cultivar. However, the results presented here are based on one season and one location experiment and need to be confirmed through further on-farm research under various soil and agro-climatic conditions. Therefore, attention shall be given to the following issue for future research:

✔ Conducting similar research over locations and seasons would be relevant to verify the current result and to get conclusive results for the best recommendation.
✔ Evaluating and reconfirming the present research result with different levels of NPSB rates less than and greater than 121 kg ha^-1^ along with the *Mesorhizobium* inoculants is needed under different agroecologies to reach a conclusive recommendation.
✔ The effectiveness of these commercial inoculants of chickpeas should be evaluated over the location and their relationship concerning native rhizobia population, soil fertility status, and cropping system needs further investigation.

Therefore, adding the above-recommended measures to the picture of higher production and balanced nutrition program should increase the number of local chickpea producers in the future as well as the area under cultivation using the better agronomic techniques. Increased levels of balanced chickpea nutrition would therefore, over time, lead to a significant increase in agricultural output and income.

## Data availability statement

The original contributions presented in the study are included in the article/supplementary materials, further inquiries can be directed to the corresponding authors.

## Author contributions

GN: Conceptualization, Data curation, Formal analysis, Funding acquisition, Investigation, Methodology, Project administration, Resources, Software, Supervision, Validation, Visualization, Writing – original draft, Writing – review & editing. GW: Conceptualization, Data curation, Formal analysis, Funding acquisition, Investigation, Methodology, Project administration, Resources, Software, Supervision, Validation, Visualization, Writing – review & editing. WT: Conceptualization, Data curation, Formal analysis, Funding acquisition, Investigation, Methodology, Project administration, Resources, Software, Supervision, Validation, Visualization, Writing – review & editing. TT: Conceptualization, Data curation, Formal analysis, Funding acquisition, Investigation, Methodology, Project administration, Resources, Software, Supervision, Validation, Visualization, Writing – review & editing.

## Footnotes

1 CWANRO (Cheha Woreda Agriculture and Natural Resource Office) (2020). Annual report for the year 2019/2020) (Cheha woreda, Emdeber) 2020. Unpublished.
